# Identification of acute myocardial infarction in patients with atrial fibrillation and chest pain with a contemporary sensitive troponin I assay

**DOI:** 10.1186/s12916-015-0410-8

**Published:** 2015-07-27

**Authors:** Christoph Liebetrau, Michael Weber, Stergios Tzikas, Lars Palapies, Helge Möllmann, Gerhard Pioro, Tanja Zeller, Andres Beiras-Fernandez, Christoph Bickel, Andreas M. Zeiher, Karl J. Lackner, Stephan Baldus, Holger M. Nef, Stefan Blankenberg, Christian W. Hamm, Thomas Münzel, Till Keller

**Affiliations:** Kerckhoff Heart and Thorax Center, Department of Cardiology, Bad Nauheim, Germany; DZHK (German Centre for Cardiovascular Research), partner site RheinMain, Frankfurt am Main, Germany; Department of Internal Medicine II, Hospital Darmstadt-Dieburg, Groß-Umstadt, Germany; 3rd Department of Cardiology, Aristotle University of Thessaloniki, Ippokrateio Hospital, Thessaloniki, Greece; Department of Internal Medicine II, University Medical Center, Johannes Gutenberg University, Mainz, Germany; Division of Cardiology, Department of Internal Medicine III, Goethe University Frankfurt, Frankfurt am Main, Germany; Department of General and Interventional Cardiology, University Heart Center, Hamburg, Germany; Department of Internal Medicine, Federal Armed Forces Hospital, Koblenz, Germany; Department of Laboratory Medicine, University Medical Center, Johannes Gutenberg University, Mainz, Germany; Department of Internal Medicine III, University of Cologne, Cologne, Germany; Department of Cardiology, University of Giessen, Giessen, Germany; Department of Cardiothoracic Surgery, Goethe University Frankfurt, Frankfurt am Main, Germany; DZHK (German Centre for Cardiovascular Research), partner site Hamburg, Kiel, Lübeck, Hamburg Germany

**Keywords:** Acute coronary syndrome, Myocardial infarction, Atrial fibrillation, Cardiac troponin

## Abstract

**Background:**

The introduction of modern troponin assays has facilitated diagnosis of acute myocardial infarction due to improved sensitivity with corresponding loss of specificity. Atrial fibrillation (AF) is associated with elevated levels of troponin. The aim of the present study was to evaluate the diagnostic performance of troponin I in patients with suspected acute coronary syndrome and chronic AF.

**Methods:**

Contemporary sensitive troponin I was assayed in a derivation cohort of 90 patients with suspected acute coronary syndrome and chronic AF to establish diagnostic cut-offs. These thresholds were validated in an independent cohort of 314 patients with suspected myocardial infarction and AF upon presentation. Additionally, changes in troponin I concentration within 3 hours were used.

**Results:**

In the derivation cohort, optimized thresholds with respect to a rule-out strategy with high sensitivity and a rule-in strategy with high specificity were established. In the validation cohort, application of the rule-out cut-off led to a negative predictive value of 97 %. The rule-in cut-off was associated with a positive predictive value of 88 % compared with 71 % if using the 99th percentile cut-off. In patients with troponin I levels above the specificity-optimized threshold, additional use of the 3-hour change in absolute/relative concentration resulted in a further improved positive predictive value of 96 %/100 %.

**Conclusions:**

Troponin I concentration and the 3-hour change in its concentration provide valid diagnostic information in patients with suspected myocardial infarction and chronic AF. With regard to AF-associated elevation of troponin levels, application of diagnostic cut-offs other than the 99th percentile might be beneficial.

**Electronic supplementary material:**

The online version of this article (doi:10.1186/s12916-015-0410-8) contains supplementary material, which is available to authorized users.

## Background

Acute chest pain is one of the most common causes of admission to an emergency department [1]. Introduction of newer, more sensitive troponin assays has proven to facilitate early diagnosis of an acute spontaneous myocardial infarction [2–4]. This improved discrimination relies mainly on the superior sensitivity of these assays; however, the downside is their reduced specificity such that there are a significant number of individuals with elevated troponin levels and symptoms indicative of an acute coronary syndrome (ACS) in whom no coronary cause can be identified [5].

In addition to the classical cascade of plaque rupture and subsequent thrombus formation followed by necrosis of dependent myocardium, other causes of myocardial infarction (MI), such as myocardial injury due to an imbalance of oxygen supply and demand, have been defined. According to the universal definition of myocardial infarction used worldwide [6, 7], which has recently been updated [8], the acute spontaneous MI is denoted MI type 1, whereas myocardial injury secondary to an ischemic imbalance is denoted MI type 2.

Atrial fibrillation (AF) is the most common arrhythmia in the general population, and due to changing demographics, the number of patients with AF is continuously increasing [9, 10]. A large proportion of AF patients will be hospitalized during their lifetime and many of these patients will be admitted to emergency departments for cardiac troponin testing [11, 12]. Different mechanisms have been proposed to lead to troponin elevation in individuals with AF [13–15]. With the availability of newer, more sensitive cardiac troponin assays, many of these AF patients will have elevated troponin levels without actually suffering an acute spontaneous type 1 MI [12, 16]. Additionally, AF often accompanies and complicates acute MI [17].

The primary aim of the present study was to evaluate the clinical performance of a contemporary sensitive troponin I assay in diagnosing type 1 MI in patients presenting with AF and clinical symptoms suggestive of an ACS in two large, distinct study cohorts. An additional objective was to define and validate an optimal diagnostic troponin I threshold in such a setting. Precise identification is of utmost importance in these individuals, as patients at risk with type 1 MI benefit from an early and aggressive MI-specific treatment [18] compared with individuals suffering a type 2 MI due to AF in whom a treatment targeting coronary alterations does not seem constructive.

## Methods

### Study population

The present study investigated the clinical application of a contemporary sensitive troponin I assay in patients presenting with concomitant AF and suspected ACS. To describe the optimal usage of troponin I determination in such a setting, two independent prospective cohorts were used. First, an ACS registry was used as a *derivation cohort* to define optimal troponin I thresholds for identification of MI type 1 in patients with AF experiencing chest pain. Second, these specifically calculated diagnostic cut-offs were applied to a large, multicenter, real-world population of patients presenting with suspected ACS and AF as a *validation cohort* to test for the diagnostic performance compared with the standard 99th percentile troponin I cut-off. Only individuals of the two cohorts with available investigational troponin I measurements as well as available electrocardiogram on admission were used in the present post hoc analyses.

### Derivation cohort

The Bad Nauheim ACS registry served as the derivation cohort. In this registry patients were enrolled consecutively from April 2003 until November 2006 and referred for early coronary angiography or primary percutaneous coronary intervention due to a potential ACS with an episode of chest pain within the prior 48 hours. Patients were either admitted directly by the emergency medical system or transferred from community hospitals. Medical history and data on the acute medical situation were assessed as described earlier [2]. Blood was drawn and an electrocardiogram was acquired upon admission directly before coronary angiography. All patients gave informed consent and the study was approved by the approval was obtained from the ethics board of the state of Hessen, Germany.

### Validation cohort

A large, multicenter, all-comers study was used as a validation cohort. Patients were enrolled consecutively who presented with pain suspected to be due to ACS at the chest pain unit of the Johannes Gutenberg-University Medical Centre in Mainz, the Federal Armed Forces Hospital in Koblenz, or the University Hospital Hamburg-Eppendorf in Hamburg between January 2007 and December 2008. An electrocardiogram was acquired directly upon admission, and blood was drawn at admission and after 3 hours. Data on the acute medical situation and the patients’ medical history were assessed as described earlier [4]. Participation was voluntary. All patients provided written informed consent. The study was approved by local ethics committees in either Rheinland-Pfalz or Hamburg for all three centers.

### Defining myocardial infarction and atrial fibrillation

The final diagnosis of MI was adjudicated according to the universal definition of MI [7, 8] in both cohorts as already described [2, 4]. Briefly, type 1 MI was diagnosed when there was evidence of myocardial necrosis that was consistent with myocardial ischemia together with clinical symptoms of ischemia or electrocardiographic changes indicative of new ischemia (new ST-segment or T-wave changes or new left bundle branch block) or imaging evidence of new loss of viable myocardium or detection of a culprit lesion on coronary angiography classified according to the Ambrose criteria. Myocardial necrosis was documented based on in-house troponin determination if there was at least one value above the cut-off value for 10 % imprecision of the respective conventional troponin test together with a rising or falling pattern (a change of at least 20 %) in serial in-house troponin measurements. The final diagnosis of MI type 1 was made by two independent cardiologists based on all available clinical, laboratory, and imaging findings blinded to the investigational troponin I measurements. In case of disagreement a third cardiologist was consulted. The presence of AF was determined in both cohorts based on the electrocardiogram obtained upon admission without differentiation of new onset of AF or persistent AF.

### Laboratory measurements

Routine laboratory parameters, including creatinine, were measured immediately after blood withdrawal by standardized methods in both study cohorts. Additionally, venous blood samples were collected on admission and after 3 hours in the validation cohort, processed immediately, and stored at −80 °C until assay.

In-house troponin, represented by cardiac troponin T in the derivation cohort and two study centers of the validation cohort, was measured in serum using a conventional commercial one-step electrochemiluminescence immunoassay (cTnT, Elecsys 2010, Roche Diagnostics, Mannheim, Germany). The lower detection limit of this assay is 0.01 ng/mL, the 99th percentile is <0.01 ng/mL, and the lowest concentration measurable with a coefficient of variation (CV) <10 % is 0.03 ng/mL, which was used as the diagnostic cut-off. At the third study center of the validation cohort troponin I was used as in-house troponin for adjudication of final diagnosis. A conventional troponin I assay was used (Dimension RxL TnI, Siemens Healthcare Diagnostics, Erlangen, Germany) with lower detection limit of 0.040 ng/mL and measuring range of 0.04 to 40.0 ng/mL. The 99th percentile is 0.07 ng/mL, and the 10 % CV used as the diagnostic cut-off is 0.14 ng/mL.

As investigational troponin a contemporary sensitive troponin I assay (Architect STAT troponin I, Abbott Diagnostics) was measured in both cohorts. For this assay, the level of detection is 0.01 ng/mL with a measuring range of 0.01–50.0 ng/mL, and the 99th percentile and the lowest concentration with CV of 10 % is 0.032 ng/mL [17]. This investigational troponin I was measured by experienced technical assistants blinded to patient characteristics in stored frozen samples. Treating physicians and research staff involved in enrollment of study participants were unaware of the measured investigational troponin I values.

### Statistical analyses

Continuous skewed variables are described as median and interquartile range and symmetric variables are presented as mean with standard deviation. Receiver operating characteristic (ROC) curves based on continuous troponin I levels were calculated in both cohorts.

In the derivation cohort of 90 patients with AF, optimized thresholds were computed by determining the cut-offs that maximized i) the sum of specificity and sensitivity (Youden-optimized cut-off, named “unweighted”) and those that yielded ii) 90 % sensitivity and iii) 90 % specificity, respectively. In addition we have considered the 99th percentile of the assay as cut-off. The uncertainty of choices i) – iii) is reflected by 95 % confidence intervals that were obtained nonparametrically by taking the 2.5 % and 97.5 % percentiles from 2,000 bootstrap replications of these evaluations.

These cut-offs have been applied to the validation cohort of 314 patients. Sensitivity, specificity, positive predictive values (PPVs), and negative predictive values (NPVs) for the individual patient groups were calculated by applying the different troponin I cut-off values and consecutively calculating the corresponding values from a two-by-two factorial design. Corresponding confidence intervals for all these proportions were calculated according to Clopper-Pearson.

Relative and absolute changes in the concentration between admission and after 3 hours (that is, absolute differences and differences divided by the baseline value times 100 %) in the validation cohort were regarded as new biomarkers that gave rise to analogously defined cut-offs. Empirical kernel density estimations of these absolute and relative changes have been plotted for both subgroups, MI and non-MI patients (Fig. 2), where the bandwidths have been chosen so as to provide optimal insight into the qualitative distribution of values.

A one-sided *P* < 0.05 was considered significant. All analyses were carried out using R 2.15 and 3.1.1 (R Foundation for Statistical Computing, Vienna, Austria).

## Results

### Baseline characteristics

The derivation cohort included a total of n=90 patients with AF out of n=1,574 patients presenting with suspected MI as published [2]. Of these individuals with AF, n=75 were finally diagnosed as having type 1 MI. Based on coronary angiography findings, 67 patients with type 1 MI and AF needed a percutaneous coronary intervention or a coronary artery bypass grafting.

The validation cohort consisted of n=314 patients with documented AF in the ECG obtained at presentation out of a total of n=1,818 patients presenting consecutively with symptoms suggestive of an acute spontaneous MI [19]. After diagnostic workup n=63 patients with AF were classified as type 1 MI, whereas out of the patients in whom type 1 MI was excluded n=21 had troponin values above the 99th percentile threshold. Of those 63 patients with type 1 MI, 52 needed a percutaneous coronary intervention or coronary artery bypass grafting. The median Synergy Between PCI With Taxus and Cardiac Surgery (SYNTAX) score in patients with MI type 1 was calculated with 12.25 (IQR 6–22.12).

In these 314 patients presenting with AF, data on previously known arrhythmias, based on information given by the patients, was available in 66 patients leading to a subcohort of 248 individuals with presumably new onset AF of whom 52 had the final diagnosis MI type 1.

Baseline characteristics of the derivation and validation cohorts are provided in Table 1.

**Table 1:** Baseline characteristics of the derivation and validation cohorts

	Derivation Cohort		Validation Cohort	
	n=90		n=314	
	n	Value	n	Value
Nr. of patients (n)		90		314
Male gender n (%)	90	62 (69)	314	203 (65)
Age, mean (SD), years		72 (11)		66 (11)
Cardiovascular risk factors				
Hypertension, n (%)	89	69 (78)	314	255 (81)
Dyslipidemia, n (%)	89	31 (35)	314	232 (74)
Diabetes mellitus, n (%)	89	23 (26)	314	64 (20)
Active smoker, n (%)	89	17 (19)	314	53 (17)
Obesity, n (%)	87	21 (24)	291	82 (28)
Family history, n (%)	89	6 (7)	314	95 (30)
Laboratory parameters on admission				
Troponin I, median (IQR), ng/mL	90	0.95 (0.03, 2.9)	311	0.01 (0.01, 0.03)
Troponin I > 99th percentile, n (%)	90	67 (74)	311	74 (24)
eGFR_MDRD_, mean (SD), ml/min	86	73 (25)	314	77 (22)
Clinical variables				
Systolic Blood Pressure, mean (SD), mmHg	86	133 (31)	310	144 (24)
Heart rate, mean (SD), bpm	89	84 (32)	310	77 (19)
Chest pain onset time, median (IQR), h		8.6 (2.9 15.5)		4.3 (2.0, 13.2)
Final diagnosis AMI type 1, n (%)	90	75 (83)	314	63 (20)

Data presented as number with percentage, mean with standard deviation (SD), or median with interquartile range (IQR) as appropriate. Obesity is defined as body mass index above 30 kg/m^2^. eGFR_MDRD_ denotes estimated glomerular filtration rate using the abbreviated modification of diet in renal disease formula

### Determination of troponin I cut-off

**Fig. 1:**
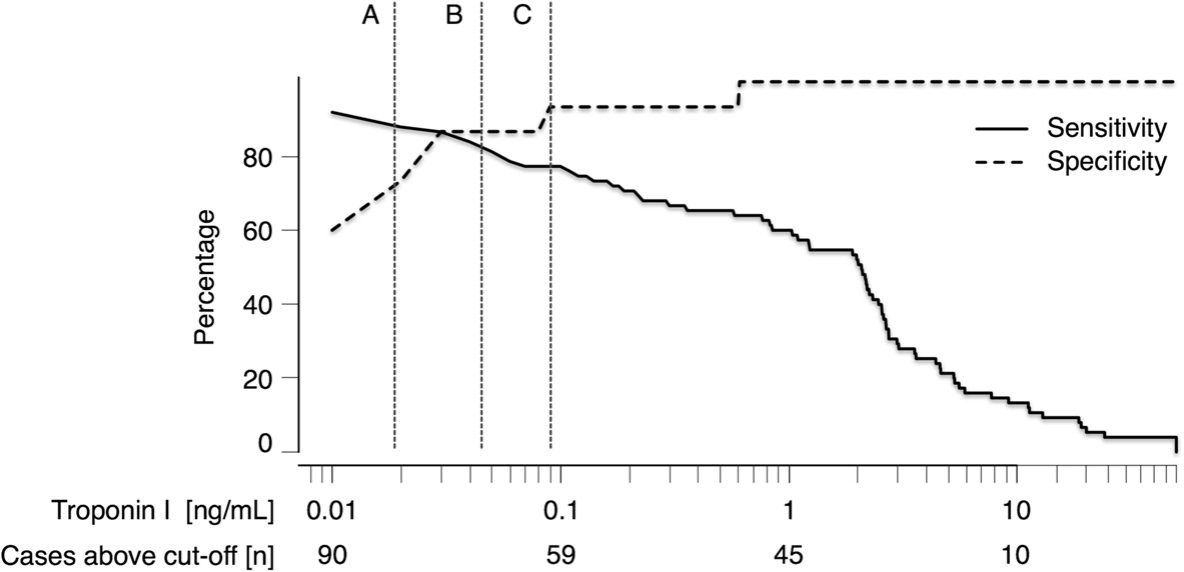
Sensitivity and specificity of troponin I determined in the derivation cohort of 90 patients with suspected acute coronary syndrome and chronic atrial fibrillation to identify patients with acute spontaneous type 1 MI. The X-axis is presented on a logarithmic scale. Lines represent different troponin I thresholds associated with 90% sensitivity (A; 0.019 ng/ml); 90% specificity (C; 0.09 ng/mL) or with the highest sum of sensitivity and specificity (B; 0.04 ng/ml) for identification of patients with acute spontaneous type 1 MI

Use of a single contemporary sensitive troponin I determination upon presentation to the emergency department in patients with AF and symptoms suggestive of an ACS of the derivation cohort yielded an area under the curve (AUC) in the ROC analyses of 0.905 (95 % confidence interval 0.841–0.970) for identification of type 1 MI. Based on these ROC analyses and using an unweighted approach by maximizing the Youden index led to an optimized diagnostic threshold of 0.04 ng/mL (95 % confidence interval 0.02–0.1 ng/mL) to identify type 1 MI. For a rule-out strategy the cut-off associated with a sensitivity of 90 % was calculated to be 0.019 ng/mL (95 % confidence interval 0.01–0.036 ng/mL). Regarding valid rule-in of type 1 MI, the optimal cut-off associated with a specificity of 90 % was calculated at 0.09 ng/mL (95 % confidence interval 0.02–0.61 ng/mL). Figure 1 presents the sensitivity and specificity for identification of type 1 MI with the corresponding potential diagnostic thresholds for identification of type 1 MI in the derivation cohort.

### Troponin I cut-off application

Application of the three different diagnostic thresholds that were calculated based on the derivation cohort with respect to overall performance (0.04 ng/mL), rule-out (0.019 ng/mL), and rule-in (0.09 ng/mL) as well as the proposed 99th percentile cut-off of the assay (0.032 ng/mL) in a large, real-world validation cohort of patients presenting with acute chest pain or equivalent symptoms and chronic AF led to the diagnostic performance presented in Table 2. If we restrict these analyses of the diagnostic performance of different thresholds to patients with presumably new onset AF, comparable diagnostic performance is observed (Additional file 1: Table S1). Furthermore, this discriminatory information of TnI to identify MI type 1 in patients presenting with AF was independent of parameters that might influence ischemic myocardial injury such as blood pressure, heart rate, and new onset of AF, as well as cardiovascular risk factors (Additional file 2: Table S2).

**Table 2:** Diagnostic performance of troponin I measured upon admission in the validation cohort

Troponin I threshold optimized for	Cut-off	Sensitivity	Specificity	PPV	NPV
	[ng/mL]	(95% CI)	(95% CI)	(95% CI)	(95% CI)
Sensitivity	0.019	0.90 (0.80-0.96)	0.88 (0.84-0.92)	0.66 (0.55-0.76)	0.97 (0.94-0.99)
Specificity	0.09	0.69 (0.56-0.80)	0.98 (0.95-0.99)	0.88 (0.75-0.95)	0.93 (0.89-0.96)
Unweighted	0.04	0.82 (0.70-0.91)	0.92 (0.88-0.95)	0.73 (0.61-0.83)	0.95 (0.92-0.98)
99th percentile threshold	0.032	0.85 (0.74-0.93)	0.91 (0.87-0.94)	0.71 (0.59-0.81)	0.96 (0.93-0.98)

Cut-offs applied are derived from the derivation cohort optimized with respect to high sensitivity and high specificity or are unweighted compared with the 99th percentile cut-off of the assay used. 95 % CI denotes 95 % confidence interval

This approach of different diagnostic thresholds relating to different clinical strategies improved potential rule-in with an increase of the PPV from 0.71 to 0.88 (*P* < 0.001) if comparing the 99th percentile cut-off and the specificity-optimized cut-off. Concerning rule-out, a slight increase in the NPV from 0.96 to 0.97 (*P* = 0.169) if comparing the 99th percentile cut-off and the sensitivity-optimized cut-off was observed.

### Addition of serial troponin I determination

A second troponin I determination 3 hours after admission was associated with an NPV of 100 % if the sensitivity-optimized, the unweighted, or the 99th percentile cut-off was used, whereas a PPV of only 82 % was achieved if the specificity-optimized cut-off was used compared with 68 % for the unweighted and 64 % for the 99th percentile cut-offs. Data on the diagnostic performance of all different thresholds calculated and the 99th percentile cut-off applied to troponin I levels determined 3 hours after admission in the validation cohort are presented in Additional file 3: Table S3.

**Fig. 2:**
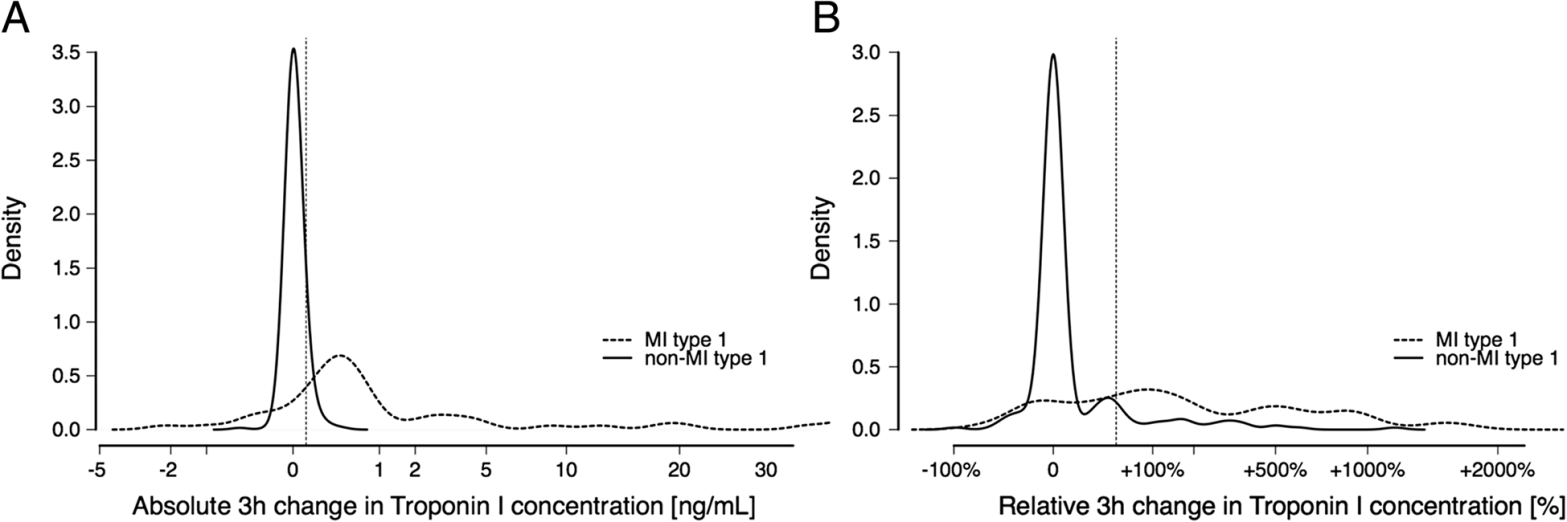
Absolute (**a**) and relative (**b**) changes in troponin I concentration within 3 hours after admission in the validation cohort in patients with atrial fibrillation with or without acute spontaneous type 1 MI. The X-axis is presented on a logarithmic scale. Dotted vertical lines represent absolute or relative troponin I changes associated with 90 % specificity for identification of an acute spontaneous MI. The X-axis is truncated

Troponin I kinetics represented by absolute and relative changes in troponin I concentration within the first 3 hours after admission are visualized in Fig. 2 with respect to final diagnosis of type 1 MI. Absolute and relative change in troponin I concentration 3 hours after admission yielded an AUC of 0.846 (95 % confidence interval 0.752–0.941) and 0.815 (95 % confidence interval 0.706–0.904), respectively, to identify patients with acute spontaneous MI.

In respect to a clinical application, based on these data concerning changes in troponin I concentration, an unweighted optimized diagnostic threshold of 0.011 ng/mL (95 % confidence interval 0.004–0.019 ng/mL) for the absolute change and of 0.3 % (95% confidence interval 0.3 %–60.3 %) for the relative change was calculated via ROC analyses. Optimization for specificity (90 %) leads to a 0.023 ng/mL (95 % confidence interval 0.013–0.123 ng/mL) absolute change and a 40 % (95 % confidence interval 30 %–150 %) relative change threshold.

The diagnostic performance of these different absolute and relative changes in troponin I concentration within 3 hours alone and in combination with troponin I determined directly upon admission is presented in Tables 3 and 4. In patients with troponin I above the 99th percentile upon admission, application of the specificity-optimized 3-hour change threshold led to a PPV of 86 % for either absolute change or relative change. In patients with troponin I levels above the specificity-optimized threshold upon admission, use of the specificity-optimized 3-hour change criterion yielded a PPV of 96 % for absolute change and of 100 % for relative change.

**Table 3:** Diagnostic performance of absolute 3-hour change in troponin I concentration

	Cut-off	Sensitivity	Specificity	PPV	NPV
	[ng/mL]	(95 % CI)	(95 % CI)	(95 % CI)	(95 % CI)
Absolute 3-h change in troponin I concentration					
*n=298, n=54 patients with MI type 1*					
*Absolute 3-h change threshold optimized for:*					
Sum of sensitivity and specificity	0.011	0.83 (0.71-0.92)	0.90 (0.85-0.93)	0.64 (0.52-0.75)	0.96 (0.93-0.98)
Specificity	0.023	0.81 (0.69-0.91)	0.94 (0.91-0.97)	0.76 (0.63-0.86)	0.96 (0.92-0.98)
Absolute 3-h change in troponin I concentration and troponin I on admission above 99th percentile cut-off					
*n=66, n=46 patients with MI type 1*					
*Absolute 3-h change threshold optimized for:*					
Sum of sensitivity and specificity	0.011	0.80 (0.66-0.91)	0.55 (0.32-0.77)	0.80 (0.66-0.91)	0.55 (0.32-0.77)
Specificity	0.023	0.80 (0.66-0.91)	0.70 (0.46-0.88)	0.86 (0.72-0.95)	0.61 (0.39-0.80)
Absolute 3-h change in troponin I concentration and troponin I on admission above unweighted optimized cut-off					
*n=61, n=44 patients with MI type 1*					
*Absolute 3-h change threshold optimized for:*					
Sum of sensitivity and specificity	0.011	0.80 (0.65-0.90)	0.65 (0.38-0.86)	0.85 (0.71-0.94)	0.55 (0.32-0.77)
Specificity	0.023	0.80 (0.65-0.90)	0.82 (0.57-0.96)	0.92 (0.79-0.98)	0.61 (0.39-0.80)
Absolute 3-h change in troponin I concentration and troponin I on admission above specificity-optimized cut-off					
*n=41, n=36 patients with MI type 1*					
*Absolute 3-h change threshold optimized for*					
Sum of sensitivity and specificity	0.011	0.75 (0.58-0.88)	0.80 (0.28-0.99)	0.96 (0.82-1)	0.31 (0.09-0.61)
Specificity	0.023	0.75 (0.58-0.88)	0.80 (0.28-0.99)	0.96 (0.82-1)	0.31 (0.09-0.61)

**Table 4:** Diagnostic performance of relative 3-hour change in troponin I concentration

	Cut-off	Sensitivity	Specificity	PPV	NPV
	[%]	(95 % CI)	(95 % CI)	(95 % CI)	(95 % CI)
Relative 3-h change in troponin I concentration					
*n=298, n=54 patients with MI type 1*					
*Absolute 3-h change threshold optimized for:*					
Sum of sensitivity and specificity	0.3	0.85 (0.73-0.93)	0.80 (0.75-0.85)	0.49 (0.38-0.59)	0.96 (0.92-0.98)
Specificity	40	0.74 (0.60-0.85)	0.88 (0.83-0.92)	0.58 (0.45-0.70)	0.94 (0.90-0.97)
Relative 3-h change in troponin I concentration and troponin I on admission above 99th percentile cut-off					
*n=66, n=46 patients with MI type 1*					
*Absolute 3-h change threshold optimized for:*					
Sum of sensitivity and specificity	0.3	0.83 (0.69-0.92)	0.40 (0.19-0.64)	0.76 (0.62-0.87)	0.50 (0.25-0.75)
Specificity	40	0.70 (0.54-0.82)	0.75 (0.51-0.91)	0.86 (0.71-0.95)	0.52 (0.33-0.71)
Relative 3-h change in troponin I concentration and troponin I on admission above unweighted optimized cut-off					
*n=61, n=44 patients with MI type 1*					
*Absolute 3-h change threshold optimized for:*					
Sum of sensitivity and specificity	0.3	0.82 (0.67-0.92)	0.47 (0.23-0.72)	0.80 (0.65-0.90)	0.50 (0.25-0.75)
Specificity	40	0.68 (0.52-0.81)	0.88 (0.64-0.99)	0.94 (0.79-0.99)	0.52 (0.33-0.71)
Relative 3-h change in troponin I concentration and troponin I on admission above specificity-optimized cut-off					
*n=41, n=36 patients with MI type 1*					
*Absolute 3-h change threshold optimized for:*					
Sum of sensitivity and specificity	0.3	0.78 (0.61-0.90)	0.60 (0.15-0.95)	0.93 (0.78-0.99)	0.27 (0.06-0.61)
Specificity	40	0.61 (0.43-0.77)	1 (0.36-1)	1 (0.78-1)	0.26 (0.09-0.51)

Optimized cut-offs associated with 90 % specificity or with highest sum of sensitivity and specificity were applied as thresholds for absolute and relative 3-hour change in troponin I concentration

Cut-offs applied to troponin I values determined upon admission were derived from the derivation cohort optimized with respect to high specificity or highest sum of sensitivity and specificity as well as the 99th percentile cut-off of the assay used. 95 % CI denotes 95 % confidence interval. MI denotes myocardial infarction

## Discussion

The availability of robust, sensitive cardiac troponin assays has been shown to substantially improve the timely diagnostic workup [3, 4] as well as outcome [20] in patients with suspected MI. This amelioration is mainly driven by the superior assay sensitivity, which is accompanied by a loss in specificity. Several disease entities other than type 1 MI have been described to be associated with elevated cardiac troponin levels. This includes patients presenting to an emergency department with a primary diagnosis of AF [21] in whom troponin I has been shown to have only a moderate positive predictive value regarding an underlying relevant coronary obstruction when using standard troponin cut-offs. Considering that a relevant proportion of patients presenting with AF also complain of chest pain and/or dyspnea [22] and that AF often accompanies and complicates acute MI [17], data concerning diagnostic troponin use in patients with chronic AF and suspected type 1 MI are urgently needed.

Based on the subgroup of patients with chronic AF taken from an intermediate- to high-risk ACS cohort, we defined potential diagnostic troponin I thresholds. The optimized cut-off to discriminate patients with type 1 MI of 0.04 ng/mL was slightly higher compared with the 99th percentile cut-off of 0.032 ng/mL. Additionally, as a major concern regarding the influence of AF is loss in specificity, we calculated a troponin I concentration that was associated with 90 % specificity that might facilitate *rule-in.* Measurement of troponin I on admission and application of the 99th percentile thresholds in 314 patients with chronic AF taken from a large, multicenter, real-world cohort of patients with suspected ACS led to the expected lower specificity with a positive predictive value of 71 % compared with 80.9 % [19] if using the whole cohort of 1,818 patients. To validly *rule in* patients, use of the calculated specificity-optimized threshold improved this positive predictive value to 88 %. Concerning *rule-out* of type 1 MI, troponin I determination 3 hours after admission was associated with a negative predictive value of 100 % if using the 99th percentile cut-off or an optimized threshold. Based on the assumption that myocardial necrosis is associated with a larger increase in cardiac troponin compared with troponin release due to ischemic imbalance, use of the change in troponin concentration should further facilitate diagnostic discrimination. This is supported by our data showing that patients with type 1 MI have larger changes in troponin I concentration within 3 hours after presentation compared with patients without type 1 MI (Fig. 2). This translates into an improvement of the positive predictive value of more than 95 % with a 3-hour change criterion of 40 % in combination with the use of an optimized diagnostic troponin I threshold upon admission.

In incorporating these results into a diagnostic algorithm, one has to bear in mind that, regardless of the presence of a type 1 MI, elevated cardiac troponin is a strong predictor of worse outcome in various settings. It was recently described that a relevant proportion of stable AF patients present with elevated troponin T [23] and I [24] levels if these are determined with sensitive assays. In these patients, elevated cardiac troponin is associated with risk of stroke, cardiac death, and major bleeding. On the other hand, the risks posed to patients of MI-specific therapy, especially dual antiplatelet therapy and oral anticoagulation, both of which are associated with increased risk of bleeding [25], underlines the need to precisely identify type 1 MI and also safely exclude patients who do not need antiplatelet drugs.

Therefore, we propose the following diagnostic procedure in patients with AF and symptoms resembling those of an acute type 1 MI. First, to safely and validly identify or exclude a type 1 MI, troponin should be sequentially determined upon admission and after 3 hours, as is also recommended by the guidelines on non-ST elevation myocardial infarction of the European Society of Cardiology (ESC) [26] irrespective of the presence of AF. Second, regarding a safe rule-out strategy, a troponin I concentration below the 99th percentile cut-off obtained 3 hours after admission is associated with a negative predictive value of 100 %, which is also in line with the ESC guidelines [26]. Third, regarding rule-in, application of specificity-optimized cut-off values higher than the 99th percentile concentration can improve the positive predictive value. Additional use of the 3-hour change in troponin I concentration further improves specificity and leads to a positive predictive value of more than 95 %, potentially facilitating identification of patients who should be treated for type 1 MI with all the consequences. Fourth, given the strong association of cardiac troponin with outcome, irrespective of an acute MI, individuals with AF and symptoms suggestive of a type 1 MI presenting on admission with troponin I levels higher than a sensitivity-optimized and lower than a specificity-optimized diagnostic threshold should be considered as patients at risk and receive an adequate diagnostic workup. Such a stepwise diagnostic approach, especially the aspects concerning the troponin I gray zone and the facilitated rule-in, seems feasible based on presented and published data; however, this needs to be tested prospectively in future studies for further validation.

Several limitations should be considered. The final diagnosis of type 1 MI, aside from being based on clinical parameters and imaging data, is based on serial troponin measurements in line with the universal definition of MI; considering the influence of AF on troponin levels, this might introduce a bias. Further studies, such as those using magnetic resonance imaging to define the diagnosis of MI according to loss of myocardial tissue, might therefore further elucidate the value of troponin testing in patients with AF and suspected MI. Several patients in the derivation cohort have been transferred from a tertiary hospital leading to higher median time between onset of symptoms and presentation which might have an influence on troponin concentrations upon admission and therefore on threshold calculation. In contrast, the high percentage of coronary angiography-confirmed type 1 MI in the derivation cohort demonstrates the low percentage of other causes of troponin elevation by parameters influencing ischemic myocardial injury. These are important aspects which need to be reflected by interpreting the data.

In addition, the proportion of MI in the validation cohort is comparable to those of other European all-comer studies but higher than those in non-European cohorts, which might limit the generalizability of the result.

## Conclusions

The use of troponin I assayed upon admission and after 3 hours to obtain data on troponin kinetics provides valid diagnostic information in patients with suspected acute spontaneous myocardial infarction and chronic atrial fibrillation. Considering the higher troponin I levels observed in patients with atrial fibrillation, application of diagnostic cut-offs other than the 99th percentile might be beneficial.

## Additional files

Additional file 1: Table S1. Diagnostic performance of troponin I measured on admission in the subcohort of 248 patients of the validation cohort with presumably new onset atrial fibrillation and suspected acute myocardial infarction type 1. Additional file 2: Table S2. Univariate and multivariate association of troponin I, clinical variables and cardiovascular risk factors, and the presence of MI type 1 in patients presenting with atrial fibrillation and symptoms suggestive of an acute myocardial infarction. Additional file 3: Table S3. Diagnostic performance of troponin I measured 3 hours after admission in the validation cohort of 314 patients with suspected acute spontaneous myocardial infarction and atrial fibrillation.

## Abbreviations

ACS: acute coronary syndrome
AF: atrial fibrillation
AUC: area under the curve
CV: coefficient of variation
MI: myocardial infarction
NPV: negative predictive value
PPV: positive predictive value
ROC: receiver operating characteristic
