# Supplementary material for: Identification of acute myocardial infarction in patients with atrial fibrillation and chest pain with a contemporary sensitive troponin I assay
Source: BMC Med. 2015 Jul 27;13:169. doi: 10.1186/s12916-015-0410-8 (PMC4515912; doi:10.1186/s12916-015-0410-8)
Supplement: Additional file 1: Table S1. — Diagnostic performance of troponin I measured on admission in the subcohort of 248 patients of the validation cohort with presumably new onset atrial fibrillation and suspected acute myocardial infarction type 1. [file 12916_2015_410_MOESM1_ESM.doc]

## *Supplementary Table 1:* Diagnostic performance of troponin I measured on admisison in the sub-cohort of 248 patients of the validation cohort with presumably new onset atrial fibrillation and suspected acute myocardial infarction type 1.

| *Troponin I threshold optimized for* | **Cut-off**  [ng/mL] | **Sensitivity**  (95% CI) | **Specificity**  (95% CI) | **PPV**  (95% CI) | **NPV**  (95% CI) |
| --- | --- | --- | --- | --- | --- |
| Sensitivity | 0.019 | 0.92  (0.81-0.98) | 0.88  (0.82-0.92) | 0.66  (0.54-0.77) | 0.98  (0.94-0.99) |
| Specificity | 0.09 | 0.71  (0.56-0.83) | 0.97  (0.93-0.99) | 0.86  (0.71-0.95) | 0.93  (0.88-0.96) |
| Unweighted | 0.04 | 0.82  (0.69-0.92) | 0.92  (0.87-0.95) | 0.72  (0.59-0.83) | 0.95  (0.91-0.98) |
| 99th percentile threshold | 0.032 | 0.86  (0.74-0.94) | 0.9  (0.85-0.94) | 0.7  (0.57-0.81) | 0.96  (0.92-0.98) |

Cut-offs applied here were derived from the derivation cohort optimized with respect to high sensitivity and high specificity or unweighted compared with the 99^th^ percentile cut-off of the assay used. 95% CI denotes 95% confidence interval.
