# Supplementary material for: Identification of acute myocardial infarction in patients with atrial fibrillation and chest pain with a contemporary sensitive troponin I assay
Source: BMC Med. 2015 Jul 27;13:169. doi: 10.1186/s12916-015-0410-8 (PMC4515912; doi:10.1186/s12916-015-0410-8)
Supplement: Additional file 2: Table S2. — Univariate and multivariate association of troponin I, clinical variables and cardiovascular risk factors, and the presence of MI type 1 in patients presenting with atrial fibrillation and symptoms suggestive of an acute myocardial infarction. [file 12916_2015_410_MOESM2_ESM.doc]

## *Supplementary Table 2:* Univariate and Multivariate Association of Troponin I , clincial variables and cardiovascular risk factors and the presence of MI type 1 in patients presenting with atrial fibrillation and symptoms suggestive of an acute myocardial infarction.

|  | ***Univariate Analysis*** | | | ***Multivariate Analysis**** | | |
| --- | --- | --- | --- | --- | --- | --- |
|  | ***ß*** | ***95% CI*** | ***p-value*** | ***ß*** | ***95% CI*** | ***p-value*** |
| Troponin I | 0.162 | ( 0.145 , 0.178 ) | < 0.001 | 0.159 | ( 0.143 , 0.176 ) | < 0.001 |
| Heart rate | 0.002 | ( 0 , 0.005 ) | 0.038 | 0 | ( -0.001 , 0.002 ) | 0.889 |
| Systolic blood pressure | 0 | ( -0.002 , 0.002 ) | 0.911 |  |  |  |
| Diastolic blood pressure | 0 | ( -0.003 , 0.004 ) | 0.834 |  |  |  |
| eGFR | -0.001 | ( -0.003 , 0.001 ) | 0.193 |  |  |  |
| Known AF vs. presumably new AF | -0.024 | ( -0.118 , 0.071 ) | 0.625 |  |  |  |
| **Cardiovascular risk factors** |  |  |  |  |  |  |
| Hypertension | 0.08 | ( -0.033 , 0.194 ) | 0.167 |  |  |  |
| Dyslipidemia | 0.007 | ( -0.094 , 0.109 ) | 0.885 |  |  |  |
| Diabetes | -0.016 | ( -0.127 , 0.094 ) | 0.77 |  |  |  |
| Smoking | 0.167 | ( 0.05 , 0.284 ) | 0.005 | 0.058 | ( -0.023 , 0.139 ) | 0.161 |
| Obesity | -0.062 | ( -0.164 , 0.041 ) | 0.241 |  |  |  |
| Family History | -0.016 | ( -0.113 , 0.081 ) | 0.746 |  |  |  |

* Only predictors with p<0.05 in the univariate analyses entered the multivariate model. eGFR denotes estimated glomerular filtration rate. AF denotes atrial fibrillation.

**Supplementary Statistical Methods:**

The ability of troponin I, heart rate, blood pressure, eGFR, previoulsy known AF and cardiovascular risk factors to predict MI type 1 has been assessed by calculation of logistic regression models, each equipped with one of the parameters in question as predictor, where the coefficient assigned to this predictor has been checked for significance. Those parameters with p < 0.05 have then been entered into a multivariable logistic regression model, with significance checked likewise.
