# Supplementary material for: Identification of acute myocardial infarction in patients with atrial fibrillation and chest pain with a contemporary sensitive troponin I assay
Source: BMC Med. 2015 Jul 27;13:169. doi: 10.1186/s12916-015-0410-8 (PMC4515912; doi:10.1186/s12916-015-0410-8)
Supplement: Additional file 3: Table S3. — Diagnostic performance of troponin I measured 3 hours after admission in the validation cohort of 314 patients with suspected acute spontaneous myocardial infarction and atrial fibrillation. [file 12916_2015_410_MOESM3_ESM.doc]

## *Supplementary Table 3:* Diagnostic performance of troponin I measured 3 h after admission in the validation cohort of 314 patients with suspected acute spontaneous myocardial infarction and atrial fibrillation.

| *Troponin I threshold optimized for* | **Cut-off**  [ng/mL] | **Sensitivity**  (95% CI) | **Specificity**  (95% CI) | **PPV**  (95% CI) | **NPV**  (95% CI) |
| --- | --- | --- | --- | --- | --- |
| Sensitivity | 0.019 | 1  (0.90-1) | 0.83  (0.77-0.87) | 0.56  (0.45-0.66) | 1  (0.97-1) |
| Specificity | 0.09 | 0.94  (0.85-0.98) | 0.96  (0.92-0.98) | 0.82  (0.70-0.91) | 0.99  (0.96-1) |
| Unweighted | 0.04 | 0.98  (0.90-1) | 0.90  (0.85-0.93) | 0.68  (0.56-0.78) | 1  (0.98-1) |
| 99th percentile threshold | 0.032 | 0.98  (0.90-1) | 0.88  (0.83-0.92) | 0.64  (0.53-0.74) | 1  (0.97-1) |

Cut-offs applied here were derived from the derivation cohort optimized with respect to high sensitivity and high specificity or unweighted compared with the 99^th^ percentile cut-off of the assay used. 95% CI denotes 95% confidence interval.
